# Immunogenicity of replication-deficient vesicular stomatitis virus based rabies vaccine in mice

**DOI:** 10.1080/01652176.2021.1930277

**Published:** 2021-06-01

**Authors:** Jung-Eun Park, Hyun-Jin Shin

**Affiliations:** a Research Institute of Veterinary Medicine, Chungnam National University, Daejeon, Republic of Korea; b College of Veterinary Medicine, Chungnam National University, Daejeon, Republic of Korea

**Keywords:** Rabies, mice, recombinant vaccine, immunogenicity, vesicular stomatitis virus, replication-deficient

## Abstract

**Background:**

Rabies is a viral disease that causes severe neurological manifestations both in humans and various mammals. Although inactivated and/or attenuated vaccines have been developed and widely used around the world, there are still concerns with regard to their safety, efficacy, and costs.

**Objective:**

As demand has grown for a new rabies vaccine, we have developed a new vesicular stomatitis viruses (VSVs) based rabies vaccine that replaces glycoproteins with rabies virus (RABV) glycoprotein (GP), or so-called VSV/RABV-GP.

**Methods:**

VSV/RABV-GP production was measured by sandwich ELISA. The generation of VSV/RABV-GP was evaluated with GP-specific antibodies and reduced transduction with GP-specific neutralizing antibodies. Virus entry was quantified by measuring the luciferase levels at 18-h post-transduction. BALB/c mice (three groups of six mice each) were intraperitoneally immunized with PBS, RABA, or VSV/RABV-GP at 0 and 14 days. At 28 days post-immunization serology was performed. Statistical significance was calculated using the Holm–Sidak multiple Student’s *t* test.

**Results:**

Mice immunized with VSV/RABV-GP produced IgM and IgG antibodies, whereas IgM titers were significantly higher in mice immunized with VSV/RABV-GP compared to inactivated RABV. The secretion profiles of IgG1 and IgG2a production suggested that VSV/RAVB-GP induces the T helper cell type-2 immune bias. In addition, the average (±SD; *n* = 3) serum neutralization titers of the inactivated RABV and VSV/RABV-GP groups were 241 ± 40 and 103 ± 54 IU/mL, respectively

**Conclusion:**

Our results confirm that VSV/RABV-GP could be a new potential vaccination platform for RABV.

## 1. Introduction

Rabies is an important zoonotic disease that cause about 55,000 human deaths annually (Hampson et al. 2015). The causative agent is a rabies virus (RABV), belonging to the genus *Lyssavirus* of the family *Rhabdoviridae* (Madhusudana et al. 2012). The single-stranded, negative-sense RNA genome of RABV encodes five structural proteins: nucleoprotein (N), phosphoprotein (P), matrix protein (M), glycoprotein (GP), and RNA-dependent RNA polymerase (L) (Albertini et al. 2011). GP represents the primary antigen of RABV and is the target for binding virus-neutralizing antibodies (Albertini et al. 2011; Zhu and Guo 2016). Neutralizing antibodies play an important role in inhibiting viral infection; according to WHO guidelines, neutralizing antibody titers higher than a serum concentration of 0.5 IU per mL are acceptable levels for protection (Moore and Hanlon 2010).

Controlling rabies in animals and mass vaccination on not only pet animals but also wildlife has been considered the most efficient strategies for protection and spread. To this end, various rabies vaccines have been developed and commercialized over the last few years (Ertl 2019; Nandi and Kumar 2010). However, there is still a need to improve characteristics, such as both the safety and targeting inhibition of transmission between animals, stability and convenient handling of the vaccine, and low costs (Amann et al. 2013).

Vesicular stomatitis virus (VSV), a member of the family *Rhabdoviridae*, has been developed as the viral vector system for expression and especially as the vaccine platform. It has already proven to be a promising attenuated vaccine vector from many studies. Recombinant VSV-based vaccines showed effective protection against variety of pathogens, such as Ebola virus, Lassa virus, Henipavirus, and thrombocytopenia syndrome virus (Marzi et al. 2015; DeBuysscher et al. 2014; Dong et al. 2019). The biggest advantage of the VSV vector system is that it is capable of growing to substantially high titers in several cell lines in vitro and elicits strong humoral and cellular responses in vivo (Zinkernagel et al. 1978).

Here, we developed a replication-deficient VSV-expressing RABV-GP as a potential rabies vaccine. We proved that a VSV-expressing RABV-GP elicited strong immune responses, similar to those of inactivated vaccines. Our study demonstrates that the VSV platform is a promising candidate for the development of a safe and efficient rabies vaccine.

## 2. Materials and methods

### 2.1. Cells and plasmids

HEK293T cells and BHK21 cells were maintained in Dulbecco’s modified Eagle’s medium (DMEM) supplemented with 10% (vol/vol) fetal bovine serum (FBS), 10 mM 4-(2-hydroxyethyl)-1-piperazineethanesulfonic acid (HEPES), 100 mM sodium pyruvate, 0.1 mM nonessential amino acids, 100 U/mL penicillin G, and 100 μg/mL streptomycin. Cell culture materials and reagents were obtained from SPL Life Sciences Co., Ltd. (Gyeonggi-do, Korea) and Gibco® BioSciences (Dublin, Ireland) unless otherwise noted.

The codon-optimized RABV-GP gene of strain Era (GenBank accession no. EF206707) was synthesized from GenScript® and cloned into the pcDNA3.1 vector. Plasmids encoding VSV-G Indiana and pHEF-VSV-G were provided by BEI Resources (Manassas, VA, USA).

### 2.2. VSV pseudotyped virus preparation and transduction

HEK293T cells were transfected with either pcDNA3.1-RABV-GP, pHEF-VSV-G, or pcDNA3.1 empty vector. For transfection, plasmid DNAs were incubated with polyethyleneimine (PEI; Polysciences, Inc., Warrington, PA, USA) at 1:3 DNA:PEI ratios in Opti-MEM (Life Technologies, Carlsbad, CA, USA) for 15 min at room temperature, and then added dropwise to the adherent cells (2 µg DNA per 10^6^ cells). At 24-h post-transfection, cells transduced with VSVluc-VSVΔG were complemented with Junin virus glycoproteins for 1 h. Cell-free supernatants were collected at 24–72 h post-transduction and filtered through a 0.45-µm filter. The pseudotyped viruses in the supernatants were pelleted by centrifugation at 10,000*g* for 10–18 h, suspended in phosphate-buffered saline (PBS, pH 7.4), and kept at −80 °C until use.

For transduction, BHK21 cells were incubated with pseudotyped viruses at normalized inputs, based on VSV M protein levels, for 1 h at 37 °C. Where indicated, mouse anti-C9 (Santa Cruz, Dallas, TX, USA) or monoclonal mouse anti-rabies virus (Rab-50, Santa Cruz, Dallas, TX, USA) were added during virus absorption. At 18-h post-transduction, cells were lysed, and luciferase levels were quantified as a measure of VSV pseudotyped virus entry.

### 2.3. Mouse immunization and sample collection

All the animal experiments were performed according to the protocol approved by the Institutional Animal Care and Use Committee of Chungnam National University (ethics approval number: CNU-01184). Eight-week-old BALB/c mice were divided into three groups of six mice each. All groups were vaccinated intraperitoneally twice at 2 weekly intervals. Mice in group 1 were given PBS and 50 μL Alum adjuvant as controls. Mice in group 2 were given 10 μg of VSV/RABV-GP and 50 μL Alum adjuvant. Mice in group 3 were given 10 μg of inactivated RABV and 50 μL Alum adjuvant. For virus inactivation, viruses were incubated with 2 mM ethyleneimine (BEI) at 37 °C overnight. The remaining BEI was neutralized by addition of 20% sodium thiosulfate. At 28-h post-initial immunizations, serum samples were collected.

### 2.4. Enzyme-linked immunosorbent assay

For sandwich enzyme-linked immunosorbent assay (ELISA), 96-well microplates were coated with mouse monoclonal anti-rabies virus antibody (RV1C5, Santa Cruz) at 1 μg/mL concentration in carbonate/bicarbonate buffer (pH 9.6) and incubated overnight at 4 °C. The plates were blocked with 5% non-fat dry milk in PBS at room temperature (RT) for 2 h and then with serially diluted VSV/RABA-GP at RT for 2 h. After four washes with PBS-T, the plates were incubated with horseradish peroxidase (HRP) conjugated anti-rabies virus (Rab-50) at RT for 1 h.

For indirect ELISA, ELISA plates were coated with inactivated RABV in carbonate/bicarbonate buffer (pH 9.6) overnight at 4 °C. The plates were blocked with 5% non-fat dry milk in PBS at RT for 2 h and then with serially diluted mouse sera at RT for 2 h. After four washes with PBS-T, the plates were incubated with HRP conjugated anti-mouse IgG, IgM, IgG1 or IgG2a at RT for 1 h. The reaction was visualized by substrate 3,3′,5,5′-tetramethylbenzidine and stopped with 1 N H_2_SO_4_. The absorbance at 450 nm was measured by ELISA plate reader.

### 2.5. Virus neutralization assays

Fluorescent antibody virus neutralization test (FAVNT) was performed as previously described (Smith et al. 1973). The serum neutralizing titers were converted and presented in international units per milliliter (IU/mL).

### 2.6. Statistical analyses

All experiments except animal experiment were independently repeated at least three times. Data are presented as mean ± SD. Statistical significance was calculated using the Holm–Sidak multiple Student’s *t* test. A *p* value of <0.05 was considered statistically significant.

## 3. Results

### 3.1. Generation and characterization of VSV/RABV-GP

**Figure 1.**
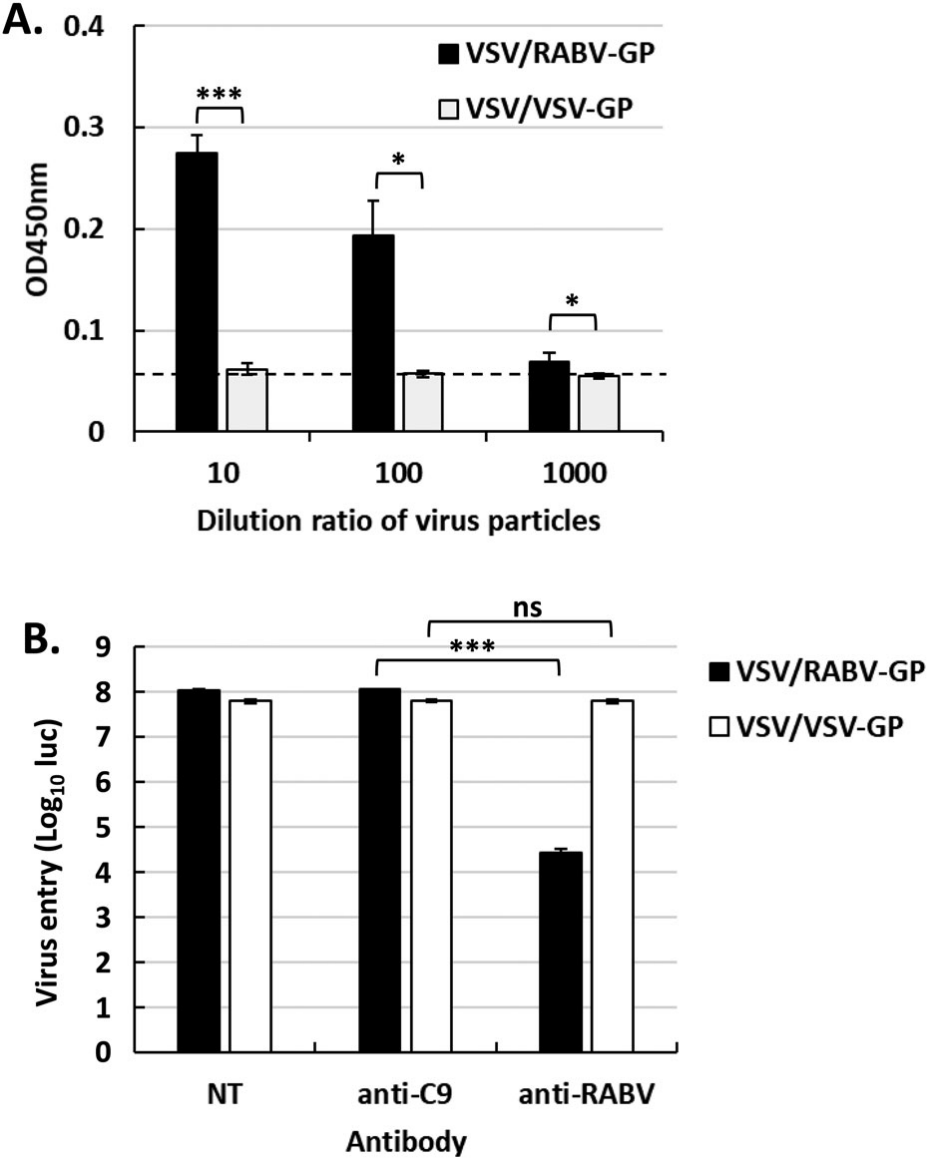
Generation and characterization of VSV/RABV-GP. A: VSV/RABV-GP production was measured by sandwich ELISA. Results have been expressed as the mean of OD450 ± SD values and are representative of at least three independent experiments. Dotted line indicates the background. B: BHK21 cells were transduced with VSV/RABV-GP or VSV/VSV-GP in the presence or absence of the indicated antibodies. Virus entry was quantified by measuring the luciferase levels at 18-h post-transduction. Error bars represent the SD from the mean (*n* = 3). Statistical significance was assessed by Student’s *t* test. *p < 0.05, ***p < 0.001; ns, not significant.

To generate VSV/RABV-GP, the GP gene of RABV from the Era strain was cloned into the pcDNA3.1 vector and is designated as pcDNA3.1-RABV-GP. The VSV/RABV-GP pseudo-typed virus was generated in HEK293T cells, as described previously (Park et al. 2016). The incorporation of RABV-GP was verified by sandwich ELISA. As shown in Figure 1(A), the absorbance at 450 nm in wells containing VSV/RABV-GP was significantly higher than that in wells containing VSV/VSV-GP or background. To evaluate whether the recombinant GP was antigenically similar to wild-type RABV-GP, the response to neutralizing antibodies in BHK21 cells was evaluated. As shown in Figure 1(B), VSV/RABV-GP transduction was significantly reduced by neutralizing antibodies, but it was not affected by the off-target antibody (anti-C9), which was used as a control. As expected, no inhibitory activity was detected against VSV/VSV-GP, the negative control. These results clearly confirmed that the RABV-GP was incorporated correctly on the surface of the VSV pseudotyped virus and displayed critical neutralizing epitopes such that it induced potential neutralizing antibodies against RABV.

### 3.2. Humoral immune responses induced by VSV/RABV-GP vaccination

**Figure 2.**
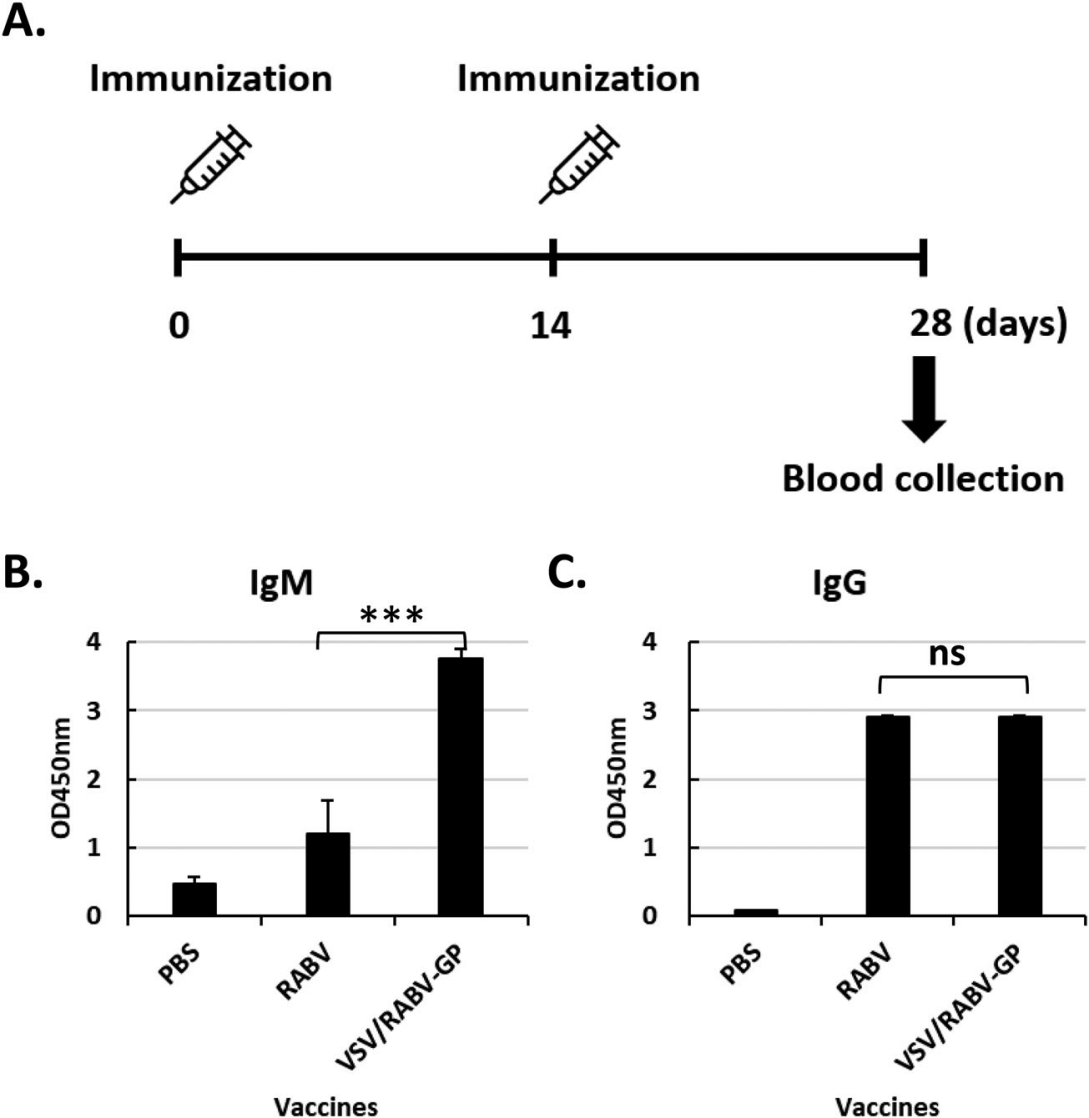
Immunization schedule and humoral immune response induced by vaccination. A: Schematic diagram of the immunization protocol. BALB/c mice were i.p. immunized with PBS, RABA, or VSV/RABV-GP at 0 and 14 days after the first immunization. At 28 days post-immunization, mice were sacrificed and serum samples were collected. B and C: Detection of the levels of RABV GP specific IgM (B) and IgG (C). Results are expressed as the mean (*n* = 6) of OD450 ± SD values and are representative of at least three independent experiments. Statistical significance was assessed by Student’s *t* test. ***p < 0.001; ns, not significant.

Next, we evaluated the immunogenicity of VSV/RABV-GP in mice. Eight-week-old BALB/C mice were intraperitoneally immunized either with VSV/RABV-GP or inactivated RABV (Figure 2(A)). None of the inoculated mice demonstrated any clinical signs or weight loss (data not shown), suggesting that VSV/RABA-GP immunization was safe as an inactivated control. On day 28 of post-immunization, IgM and IgG titers were examined. The IgM titers in mice immunized with VSV/RABV-GP were significantly three times higher than those in mice immunized with inactivated RABV (OD value 1.2 ± 0.5 and 3.8 ± 0.2, respectively [*n* = 6]), as shown in Figure 2(B). Even in the variation of IgM titer in each mouse, a higher variation was found in the RABV vaccine group, but very low variation was found in the VSV/RABV-GP group (Figure 2(B)). Comparing IgG titers, both groups showed a strong IgG titer (OD value of 2.90 ± 0.02); however, there were no statistical differences between the two groups (Figure 2(C)). We confirmed that VSV/RABV-GP vaccination induced the same level of IgG with inactivated RABV. Based on these humoral response results, we confirmed that VSV/RABV-GP induced strong antibodies of both IgG and IgM. Moreover, it induced at least the same or a higher antibody titer than inactivated RABV.

### 3.3. VSV/RABV-GP induces a T helper cell type-2 immune bias

**Figure 3.**
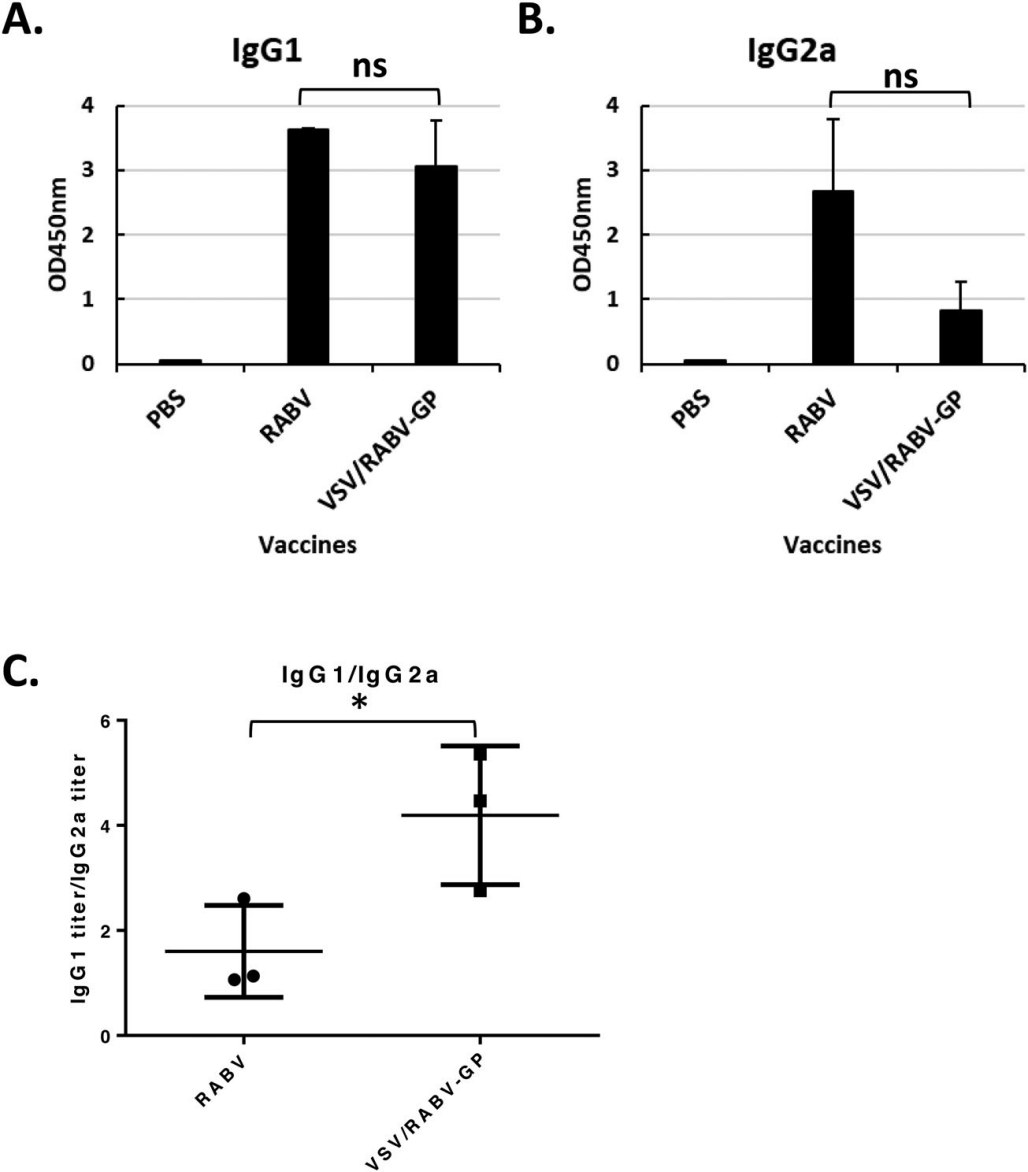
Influence of VSV/RABV-GP immunization on the type of T helper cell response. The serum specific IgG1 (A) and IgG2a (B) titers were determined as indicators of a Th2 or Th2 type of response, respectively. C: IgG1/IgG2a titer ratios of immunized mice. Results are expressed as the mean (*n* = 6) of OD450 ± SD values and are representative of at least three independent experiments. Statistical significance was assessed by Student’s *t* test. ns, not significant.

The profile of immunoglobulin isotypes is heavily influenced by the T helper cell type-1 (Th1)/ T helper cell type-1 (Th2) balance of the immune responses. The levels of IgG1 and IgG2a have been related to the Th1/Th2 predominance of immune responses (Snapper and Paul 1987; Finkelman et al. 1990). To determine whether Th1 and/or Th2 humoral response was induced by vaccination with VSV/RABV-GP, the levels of specific IgG1 and IgG2a subclasses were compared with the inactivated RABV vaccine. Sera samples showed statistically indistinguishable GP-specific IgG1 and IgG2a responses (Figure 3(A,B)). Analysis of the antigen-specific IgG1/IgG2a ratio in the sera samples revealed that mice immunized with VSV/RABV-GP had a greater IgG1/IgG2a than the inactivated RABV, namely 4.2 ± 1.3 and 1.6 ± 0.9, respectively [*n* = 3] (Figure 3(C)). The data indicate that VSV/RABA-GP induces a Th2-biased immune response.

### 3.4. VSV/RABV-GP immunization generates neutralizing antibodies

**Figure 4.**
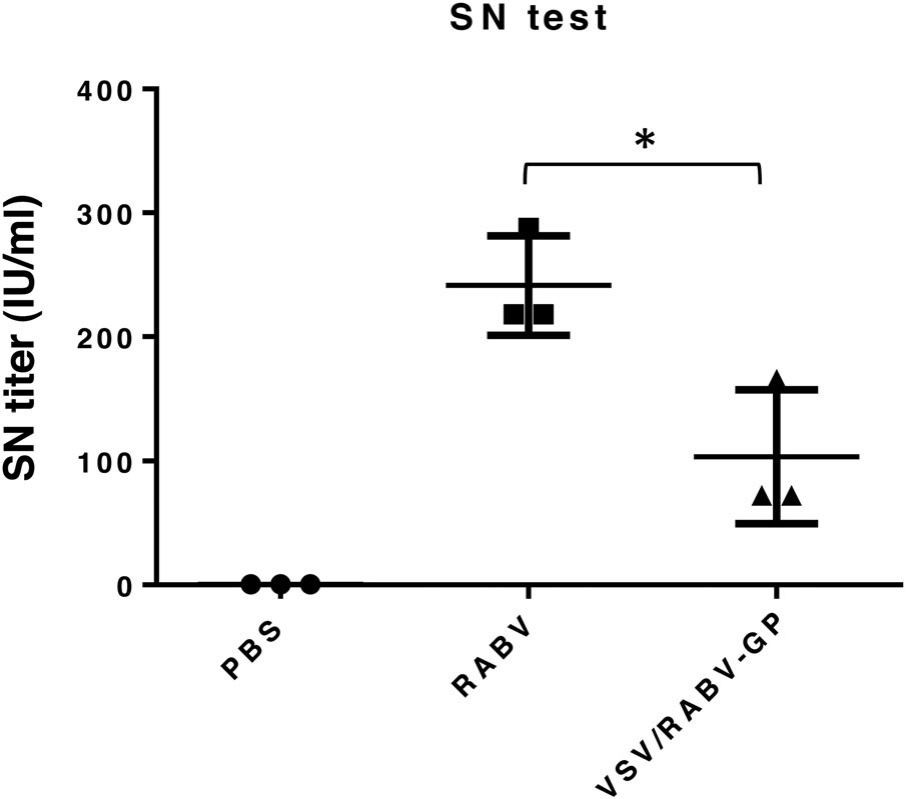
Protective efficacy of VSV/RAVB-GP vaccination. Detection of neutralizing antibody titers in the serum of the immunized mice by fluorescent antibody virus neutralization test (FAVNT). Results are expressed as the mean (*n* = 3) of serum neutralizing titers ± SD values and are representative of at least three independent experiments. Statistical significance was assessed by Student’s *t* test. *p < 0.05.

To determine whether VSV/RABV-GP can generate an immune response supporting a protective response, the neutralizing activity of the sera samples was analyzed by FAVNT. The average serum neutralization titers of the inactivated RABV and VSV/RABV-GP groups were 241 ± 40 and 103 ± 54 IU/mL, respectively (Figure 4).

## 4. Discussion

Rabies was, after smallpox, the second human disease for which an efficacious vaccine was developed, by Pasteur in 1885. Although it is eminently preventable, with highly efficacious vaccines available for both humans and animals, it still causes considerable mortality in low- and middle-income countries (Hampsom et al. 2015). Primary prevention is the best defense against rabies. It includes the following: elimination of animal rabies through dog vaccination campaigns; promoting awareness of the disease and available prevention; and encouraging responsible dog ownership and vaccination, particularly in endemic areas, where existing cultural practices might need to be adapted to integrate these interventions (Banyard et al. 2013). Where possible, rabies pre-exposure prophylaxis (PrEP) via immunization is recommended for travelers to endemic regions, although such advice might not always be followed (Fooks et al. 2003). However, in the absence of infrastructure to support such initiatives, secondary prevention attempts to prevent the onset of clinical disease through post-exposure prophylaxis (PEP). Most human cases of rabies are the result of dog bites (Fooks et al. 2017). There is a strong argument for investment in dog vaccination: the feasibility and cost-effectiveness of rabies control and human rabies elimination by dog vaccination has been demonstrated in some countries (Wang et al. 2018). Several inactivated preparations of RABV are available as vaccines to immunize humans and domestic animals. For wildlife vaccination, both live-attenuated and subunit vaccines are available (Rupprecht et al. 2016). Licensed human rabies vaccines are all based upon an inactivated rabies virus and may be used either for PrEP as well as PEP (Chulasugandha et al. 2006). PrEP regimes have until recently involved three doses of inactivated rabies virus vaccine spread over 28 days with typical costs (Chulasugandha et al. 2006). Not only inactivated vaccines, but also other recombinant vaccines trials are reported elsewhere. Recently, a simian adenovirus-vectored candidate vaccine showed that single-dose vaccination induced strong neutralizing antibodies in mice (Wang et al. 2018). Also, other rabies vaccine candidates using viral vectors have been reported (Ramya et al. 2011; Abreu-Mota et al. 2018; Chen et al. 2019; Wang et al. 2019*)*. Very recently, RNA vaccines for rabies were reported (Armbruster et al. 2019).

The importance of rabies immunoglobulin (RIG) has been studied. PEP can include both the administration of RIG and vaccination, although individuals who receive adequate PrEP should receive a booster vaccination but not RIG. The application of RIG directly to the wound aims to neutralize any replicable virus in the immediate wound area and prevent the spread of the virus in the time that it takes to develop sufficient immunity in response to vaccination.

In the present study, we generated VSV/RABV-GP and investigated its immunogenicity in mice. The results described above demonstrate that the VSV/RABV-GP immunization can elicit a strong, specific humoral immune response in mice. The average neutralizing titer in immunized mice was 103 IU/mL, which is higher than the recommended lowest titers by the WHO. VSV/RABV-GP did not induce cytotoxic effects in transduced cells or the immunized mice. Collectively, the data suggest that VSV/RABV-GP is a promising candidate for rabies vaccine.

Vaccination is considered the best way to combat a rabies infection before and after exposure, as there is no treatment available once the symptoms have appeared. Since the nerve tissue vaccine was first developed by Pasteur in 1885, various rabies vaccines for animal and human use have been developed (Nandi and Kumar 2010; Ertl 2019). Inactivated nerve tissue vaccines are economically beneficial but can cause serious side effects, such as autoimmune encephalomyelitis in inoculated animals (Cliquet and Picard-Meyer 2004). Inactivated cell cultures and embryonated egg vaccines have replaced nerve tissue vaccines and are considered safe and are well-tolerated (Kamoltham et al. 2002; Kamoltham et al. 2007*)*. However, these vaccines are expensive and unaffordable for people and animals living in developing countries. Attenuated live vaccines can efficiently elicit a protective immune response without the use of adjuvants. In contrast, there is a potential risk for rabies in the inoculated animals due to their residual virulence or pathogenic mutation during viral propagation (Nandi and Kumar 2010).

The replication-deficient VSV-vectored vaccine is a promising vaccine platform that is safe and effective. Single-cycle VSV-based vectors lacking the VSV G protein can infect cells but cannot produce infectious particles (Schnell et al. 1997). Therefore, there are no potential safety issues associated with infectious vaccines. In addition, VSV proteins have adjuvant-like properties, thereby eliciting robust adaptive immune responses to the vaccine immunogen (Cobleigh et al. 2012*;* Tober et al. 2014*;* Wu et al. 2014*)*. Nevertheless, although the VSV protein(s) is important for this enhancing effect, it has not been fully identified.

IgM possesses multiple functions that promote effective antimicrobial properties, including particle agglutination (direct neutralization), complement activation, and enhanced phagocytosis. In addition, IgM has the ability to influence the generation of adaptive immunity (Boes et al. 1998) and to influence the magnitude of virus-specific IgG antibody responses (Baumgarth et al. 2000). Natural IgM is present in the blood at high concentrations, while immune IgM is the first antibody isotype induced upon infection or immunization, suggesting that vaccine-induced IgM may help to improve the efficacy of RABV vaccination in the context of PEP (Racine and Winslow 2009). IgM plays critical roles in limiting virus dissemination for some neurotropic and non-neurotropic viruses, including West Nile virus (Diamond et al. 2003), influenza virus (Baumgarth et al. 2000; Kopf et al. 2002), and vesicular stomatitis virus (Hangartner et al. 2006). The role of IgM generated from RABV vaccination and its protection has been studied elsewhere. Dorfmeier et al. (2012) reported that antibody subtype and subclass revealed the potential for early protective IgM antibodies in recombinant RABV-M-immunized mice. One IgM antibody is needed to cover 9 or 10 glycoprotein spikes on the surface of RABV particles for neutralization, compared to one or two IgG antibodies to cover only three glycoprotein spikes (Flamand et al. 1993). Very recently, Dorfmeier et al. (2013) reported that IgM showed a very important role in postexposure vaccination. Taken together, the information from all reference studies suggests that immune IgM is induced in response to vaccination, and that the high valency and lower number of IgM than IgG needed to cover the RABV spike glycoproteins make induced IgM attractive from a PEP vaccination standpoint.

## 5. Conclusion

In conclusion, replication-deficient VSVs expressing RABV-GP displayed well-folded RABV-GP on their surface and elicited potent humoral immune responses. To our knowledge, no VSV-based vaccines against RABV have been reported. Our study provides a novel and efficient strategy for developing vaccines against this specific viral infection. However, the significant differences between mice, dogs, and humans should not be overlooked. Therefore, confirming the effectiveness and safety of vaccines in humans and dogs is an important topic for future research on vaccines against rabies. In particular, based on high level of IgM induced, our vaccine might be useful for postexposure vaccination.
